# Current Search through Liquid Biopsy of Effective Biomarkers for Early Cancer Diagnosis into the Rich Cargoes of Extracellular Vesicles

**DOI:** 10.3390/ijms22115674

**Published:** 2021-05-26

**Authors:** Irène Tatischeff

**Affiliations:** Honorary CNRS and UPMC Research Director, Founder of RevInterCell, a Scientific Consulting Service, 91400 Orsay, France; irene.tatischeff@upmc.fr; Tel.: +33-683147187

**Keywords:** extracellular vesicles (exosomes; microvesicles; oncosomes; apoptotic bodies), specific human cancers (lung; breast; colorectal; prostate; ovary; pancreatic)

## Abstract

There exist many different human cancers, but regardless of the cancer type, an early diagnosis is a necessary condition for further optimal outcomes from the disease. Therefore, efficient specific and sensitive cancer biomarkers are urgently needed. This is especially true for the cancers depicting a silent progression, and those only diagnosed in an already metastatic state with a poor survival prognostic. After a rapid overview of the previous methods for cancer diagnosis, the outstanding characteristics of extracellular vesicles (EVs) will be presented, as new interesting candidates for early cancer diagnosis in human biofluid non-invasive liquid biopsy. The present review aims to give the state-of-the-art of the numerous searches of efficient EV-mediated cancer diagnosis. The corresponding literature quest was performed by means of an original approach, using a powerful Expernova Questel *big data* platform, which was specifically adapted for a literature search on EVs. The chosen collected scientific papers are presented in two parts, the first one drawing up a picture of the current general status of EV-mediated cancer diagnosis and the second one showing recent applications of such EV-mediated diagnosis for six important human-specific cancers, i.e., lung, breast, prostate, colorectal, ovary and pancreatic cancers. However, the promising perspective of finally succeeding in the worldwide quest for the much-needed early cancer diagnosis has to be moderated by the many remaining challenges left to solve before achieving the efficient clinical translation of the constantly increasing scientific knowledge.

## 1. Introduction

Using the GLOBOCAN 2020 worldwide estimate of cancer incidence and mortality for 36 cancers in 185 countries [1], female breast cancer is the most commonly diagnosed cancer (11.7%), followed by lung (11.4%), colorectal (10%), prostate (7.3%) and stomach (5.6%) cancers. Lung cancer remained the leading cause of cancer death (18%), followed by colorectal (9.4%), liver (8.3%), stomach (7.7%) and female breast (6.9 %) cancers. Regardless of the cancer type, an early diagnosis is mandatory for the patient’s optimal outcome. Therefore, finding non-invasive efficient cancer biomarkers has been a long-lasting worldwide quest. At first, cancers were diagnosed by macroscopic symptoms and palpations of already growing tumors, when possible. Radiography and imagery brought huge improvements, but they were also concerned with progressing tumors. The appearance of some blood biomarkers, such as cancer embryonic antigen (CEA), prostate-specific antigen (PSA) and others, were interesting complementary tools for the diagnosis of some cancers. However, their poor specificity and/or sensitivity resulted in too many false positive estimates, followed by inappropriate treatments. For many years, the gold standard for establishing a final cancer diagnosis relied on tissue biopsies, but when possible, they remained quite invasive and not adapted to frequent monitoring of the cancer progression during treatment. The biggest innovative improvement came with the appearance of “liquid biopsy”, when the hallmarks of different specific cancers were searched in many human biofluids, mainly blood (serum and plasma) and urine. This search has already existed for many decades with successive and different periods, first focused on the search of circulating tumor cells (CTCs), excavated from the tumor into the blood vessels, then on circulating tumor modified nucleic acids (mainly DNAs, and after miRNAs). In the last decade, appeared a growing interest into the circulating extracellular vesicles (EVs) harboring a potentially very informative cargo upon the presence of human tumors. This review is mainly concerned with the most recent works about the EV-mediated diagnosis of human cancers.

A short description of EVs and their outstanding characteristics linked with cancer are first summarized. The final chosen references among the total references obtained by the Expernova Questel mediated search (see Section 3 and supplementary files for details), will be divided into two parts: one drawing up a picture of the current general status of EV-mediated cancer diagnosis and the second one showing recent applications of this EV-mediated diagnosis for six important human-specific cancers (lung (LC), breast, (BC), prostate (PCa), colorectal (CRC), ovarian (OC) and pancreatic (PaC)).

## 2. Outstanding Characteristics of Extracellular Vesicles (EVs) 

As previously described [2], almost all cells are increasing their influence well beyond their protecting plasma membrane by releasing many specifically conditioned EVs as intercellular communication messengers. Although the mechanisms targeting the different macromolecular and molecular components into these EVs are not yet deciphered, it is now acknowledged that EVcargoes bear a lot of information not only about their parent cells, but also about their physiological healthy or diseased state. EV research is increasing worldwide [3] and is becoming an important topic in both biology and medicine. Human cells release a wide panel of extracellular vesicles with a diameter ranging from 30 nm to 5 µm. The EV continuum is classified into three main categories, following their respective biosynthesis. Exosomes (EXs), the smallest EVs (diameter between 30 nm and 140 nm) are formed intracellularly as intraluminal vesicles along the endocytic pathway until the latest multivesicular bodies (MVBs). MVBs either fuse with lysosomes for further degradation of their obsolete contents, or some of them fuse with the plasma membrane for further releasing exosomes. Microvesicles (MVs) are directly shed from the plasma membrane as small «bubbles» containing cellular components. Apoptotic Bodies (Abs) are issued from apoptotic dying cells, which package their most important cellular contents into bigger «bubbles», also fusing with the plasma membrane and extracellularly delivering their saved cellular contents. The EV cargoes contain proteins, lipids, RNAs (mRNAs, miRNAs and other non-coding RNAs), DNAs and metabolites. However, EXs, MVs and ABs suffer from somewhat overlapping sizes and the current lack of specific biomarkers precludes any clear EV subset identification. More details about EVs are given in [4]. Previous blood microparticles (current microvesicles) attracted much attention due to their involvement in health and disease [5]. For more than two decades, exosomes have been the most studied EVs and most studies reported in this review are concerned with exosomes.

For about one decade, EVs are acknowledged as a new powerful means of intercellular communication mediating important biological functions [6]. Relative to normal cells, EV release is much increased in tumor cells. Moreover, EVs are involved in mediating tumor progression through near or distant intercellular communications [7,8]. The EV-transported miRNAs have a major influence on intercellular communication by their capacity to modify the genetic expression of the EV-recipient cells, as demonstrated by a novel mechanism of exosome-mediated genetic exchange between cells [9].

## 3. Literature Search through the Expernova Questel *Big Data* Platform

For the present review participating in the IJMS Special Issue “The Role of Extracellular Vesicles in the Diagnosis of Cancer”, the Expernova Questel database platform was used with the search term: “Cancer Diagnosis” AND (Microvesicles OR Exosomes, OR Apoptotic Bodies OR Oncosomes, OR “Extracellular Vesicles”) without any filters (see supplementary Figure S1). In “one-click”, an overview was obtained, with much information (see supplementary Figure S2 for more details), and pointing out 374 scientific papers (SPs), among which 264 were found to be either open or easily accessible through the UPMC online library. All the SPs from the ongoing search were easily exported as four Excel tables (see supplementary materials for details). From these Excel tables, corresponding Word tables were prepared by keeping for each SP, the Number, classified by decreasing year of publication, moderated by a decreasing relevance to the search as estimated by the *big data* algorithm; then, the Title, the Abstract, almost always available, the Source Name (PubMed Central, PubMed from Medline, Medline, CrossRef and Doaj), the first two mentioned Authors, the Source URL, to appreciate the SP easy Internet free access and to telecharge the corresponding pdf, and lastly the Year of publication.

The criteria for further SP selection relied mainly on the title, abstract and year of publication, followed by a complete reading of each of the corresponding telecharged pdf; thereafter, a two-part selection was performed among the 89 selected references: first, twenty-two, among the most recent ones, were selected in order to give a current insight about the ongoing studies seeking promising biomarkers for diagnosis of human cancers by liquid biopsy. This will be reported in the next part of the present review. Then, a second selection, reaching sixty-seven references, was focused on the EV-mediated diagnosis of six specific human cancers, in great need for efficient early diagnosis, and chosen for the already many studies available, generally including pilot clinical investigations. This will be detailed in Section 5.

## 4. Current Trends of EV-Mediated Cancer Diagnosis in Liquid Biopsies

For more than four decades, liquid biopsy has been explored as a non-invasive complementary tool for cancer diagnosis, however, the consideration of exosomes/EVs as quite interesting cancer biomarkers is rather new [10,11,12,13]. A short Editorial about liquid biopsy for cancer diagnosis and screening [10] recalled that “the first report of CTCs appeared in 1869” and “cfDNA released into the blood stream through cellular apoptosis or necrosis was first detected in 1948”. Although briefly mentioned as cancer biomarkers, EVs and circulating miRNAs were not yet taken into account in the optimistic assertion that “liquid biopsy holds a considerable promise for early detection of cancer”. However, at the same time, Ding et al. [11] in another Editorial considered EVs as significant biomarkers in the liquid biopsy-based cancer diagnosis and asserted that profiling EVs had the potential to improve early detection of cancers.

### 4.1. Exosomes/EVs as New Components of Liquid Biopsy

It is indeed time to consider tumor EVs and their rich cargoes as full participants in the major research efforts, which are underway for elaborating efficient non-invasive early diagnosis of cancer. Mathai et al. [12] provided a comprehensive review of the clinical utility of liquid biopsy as a complementary alternative to tissue biopsy, associated with its enumerated deficiencies. They also mentioned the advantages and disadvantages of liquid biopsy. They were especially interested in the analysis of the genetic aberrations detected in liquid biopsy, using biomarkers such as circulating cell-free DNA (cfDNAs), circulating tumor DNA (ctDNAs) and CTCs, although they also briefly mentioned that the analysis of nucleic acids in exosomes might have a potential clinical utility as a tumor biomarker in cancer. Interestingly, they illustrated the interest in liquid biopsy, mainly focusing on cfDNAs, ctDNAs and CTCs for detection of genetic alterations and early diagnosis or prognosis after treatment, in colorectal cancer (CRC), breast cancer (BC), lung cancer (LC), gastric cancer (GC) and hepatocellular cancer (HCC). Only in HCC, was the role of exosomes in the detection of mutations claimed as well established, whereas some exosomal miRNAs were considered as efficient biomarkers. Despite the huge amount of oncologic research involved in liquid biopsy, the US FDA approved only in 2016 the first liquid biopsy test for analysis of EGFR mutations in Non-Small Cell Lung Carcinoma (NSCLC) patients. However, the authors concluded that the utilization of CTCs, ctDNAs and exosomes as potential biomarkers for cancer theranostics is an emerging area with a strong potential for clinical utility. Aghamir et al. [13] also presented an overall description of liquid biopsy in which circulating tumor cells, cell-free nucleic acids (cfDNA, cfRNA, and mitochondrial cfDNA), exosomes and extrachromosomal circular DNA (eccDNA) were included and separately discussed. The presence of tumor eccDNA in blood was only suggested in 2018, whereas tumor eccDNA can be seen in approximately half of human cancers. Exosome–based liquid biopsy was defined as more homogeneous in terms of size, when compared with CTCs and cfDNAs. The use of exosomes as a predictive biomarker was said to completely rely on its protein and miRNA expression profiles. No appealing future was yet predicted for exosomes. In the conclusion, liquid biopsy was presented as a noninvasive sampling tool merely aimed to take the place of tissue biopsy or to efficiently support it.

At last, taking into account that exosomes are now considered the best biomarkers for cancer diagnosis, Chung et al. [14] provided a review of the up-to-date applications of exosomes in the diagnosis and prognosis of several diseases including cancer. First, they recapitulated the biosynthesis of exosomes released from several cell types and their identity among other EV types. Then, they discussed their main general biomedical applications, as biomarkers, drug delivery systems and therapeutic agents. Lastly, they focused on exosomes as biomarkers for various diseases including cancer, and metabolic, infectious, and neurodegenerative disorders. With regard to cancer diagnosis, they mentioned the exosome increased release from tumor cells when compared with their normal counterparts, together with their specific genomic and proteomic features usable as targets for cancer diagnostics. Moreover, they briefly recalled the main exosomal components, already used as biomarkers in some human cancers, and their biological functions. Jalian et al. [15] thoroughly reviewed the unique characteristics of exosomes as new biomarkers in early cancer detection, together with the techniques used for their isolation from body fluids and for their characterization. The authors considered the main exosomal contents, such as proteins (tetraspanins (CD9, CD63, CD81 and CD151), Rab proteins, Annexins, Flotillins, proteins involved in ESCRT complex and heat shock proteins), nucleic acids and lipids, and their individual biological functions; they summarized in two tables the studies involving, respectively, the major exosomal protein biomarkers, and the nucleic acid biomarkers in the diagnosis of different human cancers. Zhu et al. [16] summarized recent advances in various technologies for exosome isolation for cancer research. They graphically showed clinical applications of exosomes in cancer (see Figure 2 [16]). They outlined the functions of exosomes in regulating tumor metastasis, drug resistance, and immune modulation in the context of cancer development. They also reported studies involving exosomes as diagnostic and predictive biomarkers for cancer, specifically focusing on exosomal proteins and non-coding RNAs. Finally, they discussed the prospects and challenges for the clinical development of exosome-based liquid biopsies and therapeutics. Wan et al. [17] focused their review on tumor-derived exosomes (TDEs). They summarized current information on mechanisms that may differentially regulate TDE biogenesis, and mostly TDE effects on the immune system that promote tumor survival, growth and metastasis. Mounting evidence indicates that changes in cancer cells and the tumor microenvironment can regulate the biogenesis, composition, and function of TDEs. Increasing the knowledge of how TDEs differ from exosomes produced by nonmalignant cells and tissues is a necessary step for further use of TDEs in cancer diagnosis.

### 4.2. Main Exosomal Biomarkers in Liquid Biopsy for Cancer Diagnosis

Exosomes contain a huge number of macromolecular and molecular components. A first systematic study of each class of exosomal components in different cancer cell lines, compared with the ones of the corresponding normal cell lines, has pointed out many potential biomarkers for cancer diagnosis. Up to now, exosomal lipids and metabolites have been poorly investigated. The present literature search changed awareness of the current position of each exosomal biomarker for cancer diagnosis in liquid biopsy. 

#### 4.2.1. Exosomal Protein Biomarkers

The proteins were the first EV components to be investigated by means of proteomics. Recently, Bandu et al. [18] reviewed the proteome profiling of EVs and their roles in cancer biology. They recalled that EVs have been studied in relation to numerous cancers, such as colorectal, bladder, prostate, pancreatic, breast, gastric, lung, blood, ovarian, cholangiocarcinoma, hepatocellular carcinoma, and oral squamous cell carcinoma and they summarized all the cancer-specific EV proteins detected in the different studies (see Table 2 [18]). The majority of these studies revealed the relationship of cancer with changes in the protein contents of various body fluids. Moreover, they have highlighted the emerging role of EVs in cancer, specifically their role in metastasis, which opens the possibility for clinical applications in diagnosis and prognosis. Kim et al. [19] explored the key communicator role of exosomes in the cancer microenvironment through proteomics. They first specifically focused on the in vitro cancer-derived exosomal proteins in breast cancer and lung cancer, but they also considered other cancers such as colon, pancreatic and renal cancers. Then, they questioned the role of exosomal proteins in tumor microenvironment as being “friends or foes” with regard to cancer-associated fibroblasts (CAFs), T cells, natural killer (NK) cells, and tumor-associated macrophages (TAMs). They asserted that exosomal proteins from cancer cells affect the tumor microenvironment in their favour through suppressive modulation of immune cells, including NK cells, T cells and macrophages, and immune surveillance. Cancer stem cell progression and chemotherapy resistance are acquired by modulating CAFs. Together, cancer-derived exosomes and tumor microenvironment cell-derived exosomes alter the cancers to become more aggressive and able to metastasize. Among a summary of exosomal proteins selected as being candidates for further preclinical studies, they described their overall specific behavior as potential biomarkers of different cancers (see Table 3 [19]). Lastly, Sandim and Montero [20] highlighted the research of Hoshino and colleagues from several institutions, who established an unprecedented proteomic database of exomeres (non-membranous about 35 nm particles) and exosomes from human origin (named EVPs), including cell lines, tumors, and non-tumor explants, as well as different biological fluids. They demonstrated that large-scale proteomic analysis of EVPs allowed the discovery of novel EVP markers and specific signatures across different tumor types. Moreover, they demonstrated unique proteomic profiles in blood-derived EVPs across distinct cancer types. Further establishment of reliable and reproducible EVP isolation procedures and analysis protocols will be determinant to define the nanovesicle fingerprinting as the next generation for cancer diagnosis. 

#### 4.2.2. Exosomal Non-Coding microRNAs 

In 2008, following the innovative study of Valadi et al. [9], Wang et al. [21] reviewed the emerging function and clinical values of exosomal microRNAs (miRNAs) in cancer. miRNAs are the most extensively studied class of short (19–24 nts) non-coding RNAs owing to their regulatory function in gene expression. The authors explained the cell biogenesis and packaging of miRNAs into intraluminal vesicles (ILVs) of the endocytic pathway, followed by secretion of exosomal miRNAs by fusion of some multivesicular bodies (MVBs) with the plasma membrane. They clearly detailed the function of different exosomal miRNAs in cancer (see Figure 3 [21]). Briefly, in recipient tumor cells, exosomal miRNAs can control cell proliferation, invasion and metastasis. Exosomal miRNAs mediate also important special communications between tumor cells and many cells in their environment, such as cancer-associated fibroblasts (CAFs), immune cells (macrophages, T cells, and dendritic cells), and endothelial cells. They are thus playing important roles in facilitating tumorigenesis, tumor-promoting immuno-suppressive microenvironment and tumor angiogenesis. Moreover, drug-resistant tumor cells can transmit the resistant phenotype to drug-sensitive tumor cells through the horizontal transfer of exosomal miRNAs. Lastly, the authors summarized recent clinical studies, concerned with some exosomal miRNAs as potential predictive biomarkers for diagnosis and/or prognosis of many human cancers. Ingenito et al. [22] also reviewed the role of exo-miRNAs in cancer with a focus on therapeutic and diagnostic applications. Additionally, they summarized some oncogenic and onco-suppressor miRNAs and their respective increasing or decreasing influences on different tumoral characteristics, such as invasion, drug resistance, angiogenesis, growth, EMT and stemness, metastasis and apoptosis specifically associated with some human cancers. With regard to diagnosis and prognosis in cancer, they discussed the diagnostic value and the state-of-the-art of exo-miRNAs in cancer, by a survey of the available studies from 2013 to 2019 on different human cancers (non-small cell lung cancer (NSCLC), oesophageous squamous cell carcinoma, glioma, castration-resistant prostate cancer, colorectal cancer (CRC), breast cancer (BC) and ovarian cancer). From both reviews, exosomal miRNAs are emerging as promising biomarkers for cancer diagnosis.

#### 4.2.3. Exosomal Long RNAs, Including Long Non-Coding RNAs 

There are many other RNAs and the next to attract attention were long RNAs (LRs). Li et al. [23] presented an optimized strategy for exosome LR (exLR) sequencing of human plasma. The sample cohort included 159 healthy individuals, 150 patients with cancer (5 cancer types) and 43 patients with other diseases. More than 10,000 exLRs, including mRNA, circRNAs and long non-coding RNAs (lncRNAs), were reliably detected in each exLR-seq sample from 1–2 mL of plasma. Blood exLRs reflected their tissue origins and the relative fractions of different immune cell types. For each exLR-seq sample, they detected 11,952 mRNAs, 4738 circRNAs, 1022 lncRNAs and 705 pseudogenes in median. The length of the substantial fraction of human blood mRNAs ranged from 33 to 19,419 nts, with an average of 2799 nts in EVs. They further showed that the exLR profile could distinguish patients with cancer from healthy individuals, and 8 exLRs might serve as biomarkers for hepatocellular carcinoma (HCC) with high diagnostic efficiency. This study provided the first genome-wide analysis of exLRs in human blood from healthy individuals and cancer patients and showed that exLR-based liquid biopsy has the potential for cancer diagnosis. Jiang et al. [24] mainly focused on the clinical potential of circulating exosomal lncRNAs as a source of liquid biopsy biomarkers in cancer diagnosis, prognosis and response to treatment. The lncRNAs are a class of non-coding RNAs greater than 200 nts in length, which has been found closely related to the development of many types of cancer. With regard to diagnosis, they reviewed 14 studies from 2013 to 2018, dealing with eight human cancer types, and with biological specimens in serum, plasma, urine or saliva. They recapitulated the diagnostic values of 24 lncRNAs by their respective upregulated (22/24) or downregulated (2/24) expression changes. It is believed that exosomal lncRNAs are expected to become novel potential diagnostic biomarkers for early-stage cancer. Zhao et al. [25] reviewed the multiple roles of exosomal lncRNAs in cancers, whereas Zhang et al. [26] focused on the EV lncRNA-mediated crosstalk in the tumor microenvironment (TME). Briefly, lncRNAs are considered to be multifunctional. They act as decoys to attract transcription factors and influence protein expression; they regulate gene expression by recruiting chromatin modifiers to special genomic locations acting as miRNA “sponges”; they have a role in signal regulation, and they encode functional micropeptides by small open reading frames. LncRNAs can be packed into vesicles and tumor cells can secrete specific lncRNA-enriched exosomes. Dysregulated lncRNAs have been reported to be involved in regulating the proliferation, metastases and recurrence of multiple cancers, including lung cancer, prostate cancer, hepatocellular cancer, and ovarian cancer. An extensive overview of exosomal lncRNAs in cancers is given in Table 1 [25]. Moreover, lncRNAs mediate crosstalk not only between tumor cells, but also between tumor cells and stromal cells, such as mesenchymal stem cells, cancer-associated fibroblasts, endothelial cells, natural killer cells and tumor-associated macrophages. Thus, EV lncRNAs mediate the progression and chemoresistance of tumor cells in the TME as reported in Table 1 [26] for many human cancers [26]. In conclusion, EV lncRNAs appear as another valuable class of diagnostic and prognostic biomarkers in a variety of cancers.

#### 4.2.4. Exosomal Non-Coding Circular RNAs 

In 2015 a new class of noncoding RNAs, the circular RNAs (circRNAs), was found enriched and stable in exosomes [23]. The length of exonic circRNA was < 1296 nts, and the median length was 437 nts. The fraction of the back-splicing reads ratio of circRNA compared to linear splicing reads fraction was only 0.18% in peripheral blood mononuclear cells (PBMCs) but reached 4.53% in blood EVs, indicating their enrichment in blood EVs. Thus, circRNAs came on stage for becoming new cancer biomarker candidates. Wang et al. [27] reviewed the emerging function and clinical significance of exosomal circRNAs in cancer. The authors summarized the recent progress on the functional roles of exosomal circRNAs in cancer progression. CircRNAs exist in all eukaryotic cells and are generated through back-splicing, a specific form of alternative splicing, and they are more stable than linear RNAs. When enriched in exosomes, circRNAs possess a multitude of functions resulting in cancer cell proliferation, invasion, metastasis and chemoresistance, and they can exert their functions when exosomes reach neighbouring or distant cells. Although some reports mentioned that certain circRNAs act as molecular sponges for miRNAs, it seems that miRNA inhibition is not a general feature orof circRNAs. The endocytic formation and delivery of exosomal circRNAs are analogous to the ones experienced by exosomal lncRNAS in the crosstalk between the donor cell and recipient cell. The incorporation of various constituents into EVs seems to be a highly regulated process, but further studies are required to elucidate the complex mechanisms that control cargo sorting into EVs. With regard to the emerging roles of exosomal circRNAs in cancer, the high number of deregulated expressions of exosomal circRNAs in cancer patients was impressive; for example, it was reported that 1147 and 1385 exosomal circRNAs were deregulated in patients with metastatic and localized breast cancer (BCa) in comparison with healthy controls. Meanwhile, 480 exosomal circRNAs were found to be differentially expressed in metastatic BCa patients compared with patients with localized disease. The significative aberrant expression of exosomal circRNAs has been identified in various types of cancer, including gastric cancer (GC), colorectal cancer (CRC) pancreatic cancer (PC), hepatocellular cancer (HCC), cholangiocarcinoma (CCA), small-cell lung cancer (SCLC) and urogenital system tumor (see Table 2 [27]). Several pending challenges should be addressed, including the one aimed at delineating the complicated interplays among circRNAs and lncRNAs for interacting with miRNAs during cancer progression. Therefore, much work is needed before any clinical use of exosomal circRNAs is recognized as cancer biomarkers.

#### 4.2.5. Exosomal Non-Coding tRNA-Derived Small RNAs 

In 2016 appeared another family of short non-coding RNAs, as new cancer signatures. tRNA-derived small RNAs (tsRNAs), usually 18–40 nts in length are generated from the precursor of mature tRNAs. Zhu et al. [28] recalled that increasing evidence indicates that tsRNA expression is spatially and temporally controlled under physiological conditions, thus playing an important role in many biological processes. Moreover, tsRNA expression was found dysregulated in lung cancer and chronic lymphocytic leukemia. The authors demonstrated the existence of abundant tsRNAs in exosomes from cell culture media and plasma. Moreover, focusing on liver cancer, they showed that the plasma in liver cancer patients has a significantly higher tsRNA level than that in healthy controls. Notably, four tsRNAs from plasma exosomes are differentially expressed between liver cancer patients and healthy donors. This study gives the first hallmarks of the diagnostic value of tsRNAs as potential biomarkers for cancer.

#### 4.2.6. Exosomal DNAs and Other Recipient Cell Biomarkers 

With the perspective of detecting oncogenic mutations, Chennakrishnaiah et al. [29] first reviewed the different liquid biopsy platforms, each driven through the release of tumor cells and their products into biofluids. Then, they focused on EVs, more specifically on exosomes, and on membrane-less EPs such as exomeres, as carriers of diagnostic information. The authors summarized the unprecedented advantages of EVs, but also discussed some pending challenges. They also recalled the biological effects of liquid biopsy analytes, with a special interest in the oncogenic activity of liquid biopsy-associated macromolecules and their carriers (EVs/EPs). They asserted that detection of unique molecular changes occurring in the cancer cell genome and epigenome in real-real time could carry enormous therapeutic value. However, the abundance of cancer-related EVs in the blood is estimated to be low, and, moreover, EV-transported tumor-modified DNAs have been poorly studied up to now, by comparison with the many non-coding RNAs. Finally, in line with the tumor-educated platelets (TEPs) transporting RNA signatures, Rak and colleagues [29] proposed a new liquid biopsy platform, taking into account the known sequestration of mutant macromolecules by circulating phagocytes such as white blood cells, which may serve as a unique reservoir of cancer-specific biomarkers. This *leukobiopsy* remains to be experimentally and clinically tested to assess its possible diagnostic utility.

#### 4.2.7. Evaluation of the Exosomal Diagnostic Potential for Cancer

Exosomal biomarker candidates for cancer diagnosis, belonging to different EV macromolecular components, are accumulating with time and many others will probably appear, as EV-mediated liquid biopsy is still in infancy. According to the database ExoCarta (http://www.exocarta.org accessed on 24 March 2021), 9769 proteins, 3408 mRNAs, 2838 miRNAs, and 116 lipids have been identified in exosomes. Therefore, finding the best exosomal biomarkers for cancer diagnosis can be compared to looking for a needle in a haystack. However, Wong and Chen [30] brought interesting quantitative information by performing a thorough systemic review and meta-analysis to evaluate the diagnostic and prognostic potential of exosomes in patients with different types of cancer, based on currently available data. Therefore, they first identified 1233 articles in the literature by search of the PubMed database from 2010 up to September 2018, using the keywords “exosome” and “cancer” and “diagnosis” or “prognosis”. Then, finally, with strict given inclusion criteria, 56 eligible studies were included for systematic review. For assessment of diagnostic biomarkers, 47 biomarkers and 2240 patients from 30 studies were included. There were 42.6% of the biomarkers as miRNAs, followed by lncRNAs (36.2%) and proteins (19.1%). All the results concerning the 30 studies included for meta-analysis of the different exosomal biomarkers in cancer diagnosis, including all the characteristics (cancer, numbers of patients and healthy controls, exosome isolation method, and liquid biopsy sample) were gathered in Table 1 [30]. For each potential diagnosis biomarker, the receiver operating characteristics curve (ROC) was extracted together with the sensitivity and specificity. The main results were that these exosomal biomarkers had excellent diagnostic ability in various types of cancer, with good sensitivity and specificity. The parallel investigation about 50 biomarkers and 4797 patients from 42 studies showed that the exosomal biomarkers had also prognostic values in overall survival, disease-free survival and recurrence-free survival. Thus, exosomes could indeed be potential biomarkers in cancer diagnosis and prognosis.

As shown in this part of the review, the number of exosomal components candidating for being cancer biomarkers is impressively increasing and their important influence in cancer progression is also progressively deciphered. However, do they have a clinical utility? To answer this question, it was chosen to focus on six specific human cancers, with recent liquid biopsy studies involving potential EV cancer biomarkers for diagnosis. The aim of the following part is to successively review these pilot preclinical studies about, respectively, lung cancer, breast cancer, prostate cancer, colorectal cancer, ovaryovarian cancer, and pancreatic cancer.

## 5. EV-Mediated Cancer Diagnosis for Six Specific Human Cancers

### 5.1. Lung Cancer EV-Mediated Diagnosis 

Lung cancer is still the number one cause of cancer death worldwide and approximately 40% of newly diagnosed patients have distant metastasis, stressing the need for early diagnosis. In 2013, Xiang et al. [31] reviewed the extensive history of lung cancer screening from imaging in the past fifty years to innovative liquid biopsies, but challenges in early detection of lung cancer remain. Santacarpia et al. [32] provided an overview of the circulating biomarkers being evaluated for lung cancer detection, mainly focusing on results from most recent studies, the techniques developed to perform their assessment in blood and other biofluids and challenges in their clinical applications. They almost focused on the more than 80% non-small cell lung cancer (NSCLC) among lung cancers, and mostly on circulating cancer cells (CTCs), cell-free tumor-DNAs (cftDNAs) with mutations or epigenetic changes, and circulating miRNAs in the blood (serum and plasma). However, they also mentioned some studies about tumor-educated platelets (TEPs) or dealing with exosomes as promising lung cancer biomarkers. Taverna et al. [33] summarized the role of exosomes in NSCLC, stressing the importance of some associated exosomal tumor proteins reflecting the pathological processes associated with the disease. Based on an important number of clinical trials dealing with circulating miRNAs in various cancers, they selected eight miRNAs with a documented role in NSCLC and searched for their exosomal expression. They nicely advocated for the use of exosomes and sorted miRNAs as efficient liquid biopsy biomarkers in lung cancer diagnosis and prognosis; lastly, they demonstrated on stage IV clinical case, treated with Gefitinib, that isolation and characterization of exosomes in patients with NSCLC was feasible in daily practice. Fortunato et al. [34] summarized the state-of-the-art of circulating miRNAs (cf. cfmiRNAs and Exo-miRNAs) in liquid biopsy for lung cancer, focusing on the potential use of Exo-miRNAs in clinical practice. Moreover, they described the importance of exosomal miRNA cargo in lung cancer detection and discussed the analysis of circulating exosomal miRNAs in a 2013 lung cancer screening trial.

Masaoutis et al. [35] presented a very nicely documented advocacy for exosomes as a particularly promising liquid biopsy material. They first encompassed the most recent studies dealing with the role of exosomes in intercellular communication during carcinogenesis. Then, they questioned the exosome isolation methods and they recapitulated the exosomal contents as diagnostics biomarkers in NSCLC, by comparison with the many earlier studies, mostly in blood serum or plasma but also in tumor tissue biopsy, when available. Thus, they considered recent studies dealing with exosomal miRNAs and lncRNAs, exosomal proteins and exosomal nucleic acids in NSCLC diagnosis. They also summarized studies about exosomal contents as prognostic and predictive biomarkers in lung cancer liquid biopsy and even mentioned exosomes as drug delivery strategy in lung cancer management. In conclusion, they stressed that exosomes will very probably be employed from the translating research into future clinical practice, after further necessary investigations to better clarify their effects and mechanisms of action.

In the context of lung cancer, Alipoor et al. [36] studied tumor-derived exosomes (TEXs) in order to better understand the mechanisms underlying tumor metastasis and progression. They especially focused on the role of TEXs in the lung tumor cellular microenvironment and on the EV-mediated intercellular talks involved in the immune response. They also considered the role of TEXs in epithelial–mesenchymal transition (EMT) and in angiogenesis enhancement. Lastly, they reviewed a few studies, showing that TEXs have the potential to act as biomarkers both for lung cancer diagnosis and prognosis. In 2020, Hu et al. [37] summarized the recent findings on exosomal miRNAs involvement in lung cancer, i.e., cellular proliferation, angiogenesis, EMT and metastasis, drug resistance, immunity and cross-talks between tumor and non-tumor cells. They also highlighted their clinical implications as diagnostic, predictive and prognostic biomarkers in lung cancer, as well as their promising potentialities for drug delivery. However many challenges remain to be solved before efficient miRNAs clinical translation.

Some studies are still devoted to the search of the best exosomal biomarkers for lung cancer diagnosis, by modifying some parameters such as type of lung cancer, state of the disease, nature of the human biofluids for isolation of different kinds of exosomes and choice of the exosomal components.

Roman-Canal et al. [38] questioned for the first time EV-associated miRNAs from pleural lavage as potential diagnostic biomarkers in lung cancer. By analysis of the differential expression of EV-associated miRNAs from 25 control pleural fluids and 21 pleural lavages from surgical lung cancer patients, they found a list of 14 miRNAs that were significantly dysregulated; among them, three miRNA-1–3p, miRNA-144–5p and miRNA-150–5p, were found to be the most promising biomarkers of lung cancer diagnosis with an accuracy of 0.941, 0.882 and 0.912, respectively. This proof of concept investigation opens the avenue to EV-associated miRNAs of pleural fluids as an untapped source of biomarkers. Zhang et al. [39] first selected for NSCLC diagnosis a serum exosome-derived 6 miRNA panel (miR-17–5p, miR-18a-5p, miR-19a-3p, miR-19b-15p, miR-20a-5p and miR-92a-1–5p), known as miR-17–92 cluster and dysregulated in other cancers; they further demonstrated for the first time that the expression of miR-17–5p was significantly up-regulated in NSCLC patients compared with the healthy controls. By adding three conventional blood tumor markers (each with disappointing sensitivity or specificity for NSCLC diagnosis), the newly developed non-invasive diagnostic 4-molecule panel, consisting of exosomal miR-17 5p, CEA, CYFRA21–1 and SCCA, was suggested to have a considerable clinical value in the diagnosis of NSCLC, although not yet able to discriminate between adenocarcinoma (Ad) and squamous carcinoma (SCC). An et al. [40] assessed unique EV protein profiles as diagnostic biomarkers for early and advanced NSCLC. EVs were enriched from the sera of early and advanced NSCLC patients and healthy controls and from cell culture supernatants of lung adenocarcinoma and bronchial epithelial cell lines; 32 proteins were identified in a common cluster of this proteome study. Among them, six significant proteins were screened and fibronectin was selected for further in vitro studies and clinical validation, showing great potential for clinical use and demonstrating the efficacy of this method for EV-associated biomarker screening. Niu et al. [41] performed a clinical study about the exosomal expression levels of alpha-2-HS-glycoprotein (AHSG) and extracellular matrix proteins (ECM1) in the serum exosomes from healthy donors (*n* = 46) and NSCLC patients (*n* = 125). Each of the two proteins showed a diagnostic capacity, which was increased when both were combined and even more when the blood CEA test was joined. 

Fang et al. [42] searched to identify early biomarkers of lung adenocarcinoma (LA), the most commonly occurring NSCLC histological type. EVs were separated from the plasma of 153 LA patients and 75 healthy controls. A three-stage EV-derived miRNAs expression profiling identified four dysregulated miRNAs (miR-505–5p, miR-486–3p, miR-486–5p and miR-382–3p) in the plasma of LA patients. The levels of miR-505–5p, which were statistically increased in LA plasma EVs, were also significantly increased in tumor samples, compared with the corresponding adjacent normal tissues. Functional studies demonstrated that EV-miR-505–5p delivered to lung cancer cells functions as an oncogene, promoting cell proliferation and inhibiting cell apoptosis via the targeting of tumor protein P53-regulated apoptosis-inducing protein 1. EV-derived miR-505-p was identified as a candidate molecule for early-stage LA detection. 

Cao et al. [43], started from the difficulty of distinguishing the tumor-derived EVs from those released by other tissues, and hypothesized that analysing the EV-related molecules in tumor tissues would help to estimate the prognostic value of tumor-specific EVs. Thus, they established an EV-associated gene signature correlating with hypoxic microenvironment and predicting recurrence in lung adenocarcinoma. Xiao et al. [44] characterized the high-affinity peptide ligand LXY30 targeting α3β1 integrin in the detection, cellular function, imaging, and targeted delivery of in vitro and in vivo NSCLC models. This is the first report of a novel potent integrin-binding peptide LXY30, that can detect and enrich live circulating tumor cells and tumor-derived exosomes from human NSCLC cell lines, and from biofluids from patients with advanced NSCLC. Furthermore, high expression of α3 integrin was associated with a poor prognosis of lung squamous carcinoma. Peng et al. [45] explored the possibility of using plasma-derived exosomal miRNAs as potential biomarkers for optimal selection of patients with advanced EGFR/ALK-negative NSCLC to immunotherapy. They identified three potential predictors of the efficacity of immunotherapy, i.e., exosomal miRNAs (hsa-miR-320d, hsa-miR-320c and hsa-miR-320b), whose common up-regulation was correlated with unfavorable response to anti-PD-1 treatment. Furthermore, they identified one potential target in the T cell suppressor plasma exosomal hsa-miR-125b -5p-5p, which might be down-regulated during the antiPD-1 treatment, suggesting that the corresponding NSCLC patients may obtain increased T-cell function and respond well to immunotherapy.

Lastly, Dong et al. [46] developed bio-inspired NanoVilli Chips for selective enhanced capture of tumor-derived EVs from blood plasma samples. At optimal conditions, NanoVilli Chips enabled highly efficient, reproducible and rapid (30 min) isolation of tumor-derived EVs, allowing non-invasive detection of specific oncogenic gene alterations that correlates with treatment response and NSCLC disease progression. Liu et al. [47] developed a non-invasive specific detection of human sera exosomal miRNAs via tethered cationic lipoplexnanoparticles (tCLN) biochip for lung cancer diagnosis. A total of five miRNAs (miR-21, mir-25, miR-155, miR-210 and miR-486) were selected as the biomarkers. When combined, the tCNL assay distinguished normal controls from all NSCLC patients with a sensitivity of 0.969 and specificity of 0.933. It also demonstrated its great potential as a liquid biopsy assay for lung cancer early detection.

### 5.2. Breast Cancer EV-Mediated Diagnosis

Breast cancer (BC) is a very heterogeneous disease, with classification into many different types and the favorable patient’s outcomes are highly dependent upon early diagnosis. Survival for early-stage BC (stage 1) remained at 100% at 1, 3, and 5 years from diagnosis. However, survival for metastatic BC (stage 4) reduced to 69% at 1 year, 47% at 3 years, and 32% at 5 years from diagnosis [48]. The current studies for EV-mediated BC diagnosis by liquid biopsy will first be described to give an insight into the still preliminary searches, which will certainly gain interest in the near future. By far the most complete review about non-invasive biomarkers for BC early detection has been elaborated by Li et al. [48] and will be further detailed, although still rather poorly involved with EV-mediated diagnosis. 

As for other cancers, pilot studies are now proposing methods for non-invasive EV-mediated diagnosis of breast cancer, based on different components of tumor cells-derived EVs circulating in human body fluids. The general frame of these studies is first an in vitro established knowledge by means of appropriate cell cultures focusing about the interesting EV components. This is followed by a primary clinical validation of these selected EV components with a small number of patients suffering from specific cancer compared to healthy controls. If conclusive, all these pilot studies warrant further standardized large-scale multicenter validation before reaching any general clinical use. The focused tumor-related EV components may be proteins, different RNAs (mostly miRNAs and other non-coding RNAs) and less often DNAs, lipids or metabolites. 

Concerning BC, two recent studies involved exosomes. Norouzi-Barough et al. [49] summarized the recent literature on the potential role of tumor-derived exosomes (TDEs) in cancer progression and the many circulating exosomal biomarkers already suggested as candidates for non-invasive liquid biopsy diagnosis of breast cancer. You et al. [50] claimed the potential of label-free optical imaging of EVs in live cells for clinical applications. Quantitative analysis of living tumor-bearing animals and fresh excised human breast tissue revealed an abundance of NAD(P)H-rich EVs within the tumor, near the tumor boundary, and around vessel structures. Levels of NAD(P)H-rich EVs were correlated with early and late-stage human BC diagnosis, whereas the percentage of NAD(P)H-rich EVs was related to their cancer status (Stage 1 (*n* = 19), Stage 2 (*n* = 21), Stage 3 (*n* = 13) and healthy (*n* = 52)). 

Three studies were concerned with EV proteins. Moon et al. [51] were involved for the first time with fibronectin (FN) present on the EV surface detected in plasma samples from disease-free individuals (*n* = 70), patients with BC (*n* = 240), patients with BC after surgical resection (*n* = 40), patients with benign breast tumor (*n* = 55), and patients with non-cancerous diseases (thyroiditis, gastritis, hepatitis B, and rheumatoid arthritis; *n* = 80). FN levels were significantly elevated (*p* < 0.0001) at all stages of BC, and returned to normal after tumor removal. Cui et al. [52] were concerned with the cancer-testis antigen lactate dehydrogenase C4 (LDH-C4). They evaluated the LDH-C4 protein expression in BC tissues and the LDH mRNA expression in serum and serum-derived exosomes of BC patients. The LDHC level in serum and exosomes could distinguish BC cases from healthy individuals, negatively correlated with medical treatment and positively with the recurrence of BC. Survival analysis showed that LDH-C4 expression negatively correlated with BC prognosis. Korolkova et al. [53] listed the diverse roles of the calcium-dependent membrane binding (AnxA6) in Triple-Negative Breast Cancer (TNBC) diagnosis and prognosis. They highlighted the potential tumor suppressor function in TNBC progression and metastasis and discussed the concept of therapy-induced expression of AnxA6 as a novel mechanism for acquired resistance of TNBC to tyrosine kinase inhibitors. Although being a predominantly intracellular protein, AnxA6 is also secreted as an EV component and confirmed in TNBC cell-derived exosomes. Moreover, AnxA6-enriched EVs from cancer-associated fibroblasts elicited proinvasive properties when taken up by breast cancer cells. The authors claimed that detection of AnxA6 might not only be useful as a potential biomarker for specific breast cancer subtypes but might also be a promising predictor of especially basal-like TNBC to targeted therapeutic interventions.

Most of the recent pilot studies about EVs-mediated BC diagnosis involved different RNA components of tumor EVs. Five of them were concerned with miRNAs. Hannafon et al. [54] pointed out that circulating exosome miRNAs were not yet well evaluated as biomarkers for breast cancer diagnosis or monitoring. They collected exosomes from the conditioned media of human breast cancer cell lines, mouse plasma of patient-derived xenograft model, and 32 human plasma samples (16 healthy and 16 from breast cancer patients). They showed that certain miRNA species, such as miR-21 and miR-1246 were selectively enriched in human breast cancer exosomes and significantly elevated in the plasma of patients with breast cancer. Li et al. [55] explored whether miRNAs from the miR-106a–363 cluster on chromosome X can be detected in the circulation of BC patients and whether these miRNAs can serve as potential diagnostic biomarkers. The expression of 12 miRNAs from the miR-106a–363 cluster was evaluated in 400 plasma samples (from 200 BC patients and 200 healthy controls (HCs)) and 406 serum samples (from 204 BC patients and 202 HCs) via a three-phase study (testing, training and external validation). The identified miRNAs were further examined in tissues (32 paired breast tissues), plasma exosomes (from 32 BC patients and 32 HCs), and serum exosomes (from 32 BC patients and 32 HCs). Upregulated levels of four plasma miRNAs and four serum miRNAs were identified and validated in BC. A plasma 4-miRNA- and a serum 4-miRNA panel were constructed to discriminate BC patients from HCs. Two overlapping miRNAs (miR-106a-5p and miR-20b-5p) were consistently upregulated in BC tissues. Four plasma miRNAs (miR-106a-3p, miR-106a-5p, miR-20b-5p, and miR-92a-2-5p) and four serum miRNAs (miR-106a-5p, miR-19b-3p, miR-20b-5p, and miR-92a-3p) from the miR-106a–363 cluster were identified as promising novel biomarkers for the diagnosis of BC. The same team [56] performed a four-phase study for profiling miRNA expression in plasma samples from a total of 257 BC patients and 257 normal controls (NCs). They identified five plasma miRNAs (let-7b-5p, miR-122-5p, miR-146b-5p, miR-210-3p and miR-215-5p) that could serve as a promising biomarker for BC detection. However, none of those miRNA biomarkers reported by other studies for BC diagnosis were identified in the present study, more involved in whole plasma miRNA biomarkers than in plasma exosomes-derived miRNAs. The remaining discrepancy between the different observations concerning the miRNA biomarkers for BC diagnosis has to be explained before any further clinical validation. Rodriguez-Martinez et al. [57] proposed the use of serum exosomal miRNAs and blood circulating tumor cells (CTCs) as a complementary clinical tool for improving BC diagnosis and prognosis in BC patients under neoadjuvant chemotherapy. Before neoadjuvant therapy, exosomal miRNA-21 and miRNA-105 expression levels were higher in metastatic versus non-metastatic patients and healthy donors. Likewise, higher levels of miRNA-222 were observed in basal-like (*p*  =  0.037) and in luminal B versus luminal A (*p*  =  0.0145) tumor subtypes. Exosomal miRNA-222 levels correlated with clinical and pathological variables such as progesterone receptor status (*p*  =  0.017) and Ki67 (*p*  =  0.05). During neoadjuvant treatment, exosomal miRNA-21 expression levels directly correlated with tumor size (*p*  =  0.039) and inversely with Ki67 expression (*p*  =  0.031). Finally, higher levels of exosomal miRNA-21, miRNA-222, and miRNA-155 were significantly associated with the presence of CTCs. Ando et al. [58] suggested a novel early-stage BC screening by the combined expression of miR-21 and matrix metalloproteinase-1 (MMP-1)/CD63 in urinary exosomes; 22 patients with relatively early detected BC were enrolled for the study; two patients were in Stage 0, seven in Stage I, seven in stage II and the remaining six in Stage III. For miR-21, the sensitivity was 0.708 and the specificity 0.792 in primary screening for BC patients against the 26 healthy controls. The sensitivity and specificity of MMP-1/CD63 expression were 0.783 and 0.840, respectively. When expression of miR-21 and MMP-1/CD63 were combined, the final sensitivity and specificity in BC screening were 95% and 79%, respectively.

One study was focused on long noncoding RNA (lncRNA) H19 in the blood circulating exosomes as a novel biomarker for BC diagnosis. Zhong et al. [59] measured the levels of lncRNA H19 in serum-derived exosomes from BC patients (*n* = 50) or patients with benign breast disease (BBD) (*n* = 50) and healthy subjects (*n*= 50). H19 levels were also measured for pre-operative and post-operative patients. Exosomal H19 expression levels were upregulated in patients with BC compared to those in patients with BBD and healthy controls. The median serum exosomal H19 levels were significantly lower in post-operative than that in the pre-operative patients. Exosomal H19 analysis had a sensitivity of 87.0% and specificity of 70.6%, which were higher than the ones for the current blood biomarkers CA153 and CEA. Moreover, exosomal H19 expression levels were associated with lymph node metastasis (*p* = 0.039), distant metastasis (*p* = 0.008), TNM stages (*p* = 0.022), ER (*p* = 0.009), PR (*p* = 0.018), and Her-2 (*p* = 0.021). Lastly, Wang et al. [60] conducted a less conventional approach, aimed at exploring whether tRNA-derived fragments (tRFs) and halves tRNA (tiRNAs) could be detected in plasma and whether they could serve as diagnostic biomarkers. A total of 316 plasma samples (176 patients with Early breast cancer (EBC) and 140 NCs) and 35 paired tissues were included in a four phases study. Thirty tRFs and tiRNAs were selected in the screening phase and then assessed in training, testing, and external validation phases. Six tRFs (tRF-Glu-CTC-003, tRF-Gly-CCC-007, tRF-Gly-CCC-008, tRF-Leu-CAA-003, tRF-Ser-TGA-001, and tRF-Ser-TGA-002) were found significantly down-regulated in plasma samples of patients with EBC compared with normal controls, and all were derived from 5’ ends of tRNAs. Patients with HER2+ EBC with low expression levels of tRF-Glu-CTC-003 were related to worse disease-free survival and overall survival. The identified tRFs were further evaluated in cell supernatants (9 BC vs. 1 NC), exosomes isolated from plasma (24 BC vs. 16 NC), and tissues (35 BC vs. 35 NC). Six plasma tRFs derived from the tRNAs 5’ ends were thus identified as promising novel diagnostic EBC biomarkers.

“The icing on the cake” of the current studies about liquid biopsy for non-invasive BC early detection is, indeed, to be found in the review from Li et al. [48]. After listing the common methods for BC screening and diagnosis, they summarized the current progress of research in non-invasive liquid biopsy focused on BC diagnosis. They first distinguished the various human biofluids potentially harboring BC biomarkers into blood-based sources (plasma, serum) and the non-blood-based body fluids, i.e., tears, breath, nipple aspirate fluid, apocrine sweat and urine. Then, they summarized the different investigated BC biomarkers, i.e., circulating tumor cells (CTCs), circulating serum carcinoma proteins (CA153, CA27-29, CA-125, CEA), circulating cell-free tumor DNAs, circulating miRNAs, different Extracellular Vesicles (EVs), and many others such as proteins, carbohydrates, lipids and metabolites. In Table 1 [48], they present a summary of all studies about non-invasive biomarkers for BC early detection, with their clinically measured sensitivity and specificity. As a conclusion, they asserted that even though the blood has been the main source for biomarker discovery, a number of very promising biomarkers have been identified from other body fluids. They also noticed that although many potential biomarkers have been reported, most of them remain in phase 1 (preclinical exploratory) or 2 (clinical assay and validation), a few in phase 3 (retrospective longitudinal) or 4 (prospective screening) and none in phase 5 (cancer control)”. Therefore, there might still be a long way for the clinical routine use of a more accurate, sensitive and cost-effective non-invasive test for early BC detection, and this is especially true for EVs, the newest appeared cancer biomarkers with many promising potentialities.

On the other hand, many microfluidic devices for exosomes isolation and analysis are currently developed with the aim of using a small volume, low cost, shorter time, and simple to operate set-up, for future clinical applications. Akagy et al. [61] proposed on-chip electrophoresis as a useful method for the differential protein expression profiling of individual EVs. Using EVs collected by MDA-MB-231 human BC cells, on-chip immunoelectrophoresis sensitively detected over-expression of CD63 glycoproteins on the surface of individual EVs. Moreover, as a proof of concept, EVs of tumor origin circulating in the blood of a mouse tumor model were also differentially detected among other EVs of non-tumor origin, showing a promising approach to the low-invasive diagnosis of cancer by liquid biopsy. Fang et al. [62] elaborated a microfluidic device that enabled on-chip immunocapture of exosomes from cancer cell culture medium and patient plasma. To capture exosomes, CD63 antibody was associated with magnetic nanoparticles (Mag-CD63). PK67 was used to detect general exosome capture. MHC-I, EpCAM and HER2 were used to detect specific exosomes. Three types of human breast carcinoma cell lines, MCF7, MDA-MB231 (M231) and SK-BR-3 were used, with primary human normal fibroblasts (NF) as a control. EpCAM-positive exosomes from MCF7 and M231 cell culture medium were significantly higher than those from NF culture medium, and this was also observed in breast cancer patients (*n* = 6) compared to healthy controls (*n* = 3) (*p* < 0.01). It suggests that the plasma exosomal EpCAM might provide diagnostic assistance for non-invasive, early detection of breast cancer. Moreover, quantification of plasma HER-2 positive exosomes, almost consistent with that in tumor tissues, allowed molecular classification of breast cancer in 19 patients, which is important for further personalized treatment. Zhang et al. [63] intended to selectively capture tumor-derived exosomes (TEX) and proposed a simple, rapid, and specific “on-off” type mucin-1 (MUC1) protein fluorescence aptasensor for detection of breast cancer. They used the device to detect exosomes derived from MCF-7, MCF-7/ADR, A549, MGC-803 and Hs578Bst cells and blood serum from female breast cancer patients (*n* = 12) and healthy donors (*n* = 10). In both cases, the breast cancer cells-derived exosomes depicted a much higher MUC1 fluorescence, indicating the potential use of this method for further diagnosis of breast cancer. Chen et al. [64] developed a new microfluidic device for immunomagnetic separation and detection of circulating exosomes in blood of breast cancer patients, able of on-chip isolation and detection of circulating exosomes within 1.5 h. A statistically significant increase (*p*  <  0.01) in EpCAM-positive exosomes was captured for breast cancer patients (*n*  =  10), when compared to healthy individuals (*n*  =  10). The device demonstrated high predicting accuracy for tumor exosomal markers with a sensitivity of 90% and a specificity of > 95%, providing a new automated platform to assist BC diagnosis. 

### 5.3. Prostate Cancer EV-Mediated Diagnosis 

Worldwide, prostate cancer (PCa) is the second most frequent cancer diagnosis and the fifth leading cause of cancer death in men. Serum prostate-specific antigen (PSA) testing has helped to increase early detection and decrease mortality from prostate cancer but suffers from poor specificity, requiring an invasive tissue biopsy for diagnosis confirmation and leading to some inappropriate overtreatments, although limited by the current active surveillance strategy. This highlights the urgent need for minimally invasive biomarkers with improved specificity and sensitivity for prostate cancer detection, diagnosis and monitoring. Liquid biopsy has been paving the way for finding such biomarkers in a patient’s body fluids, first with circulating tumor cells (CTCs) and cfDNAs, and more recently with EVs.

Recently, Lorenc et al. [65] nicely advocated the use of exosomes in PCa diagnosis, prognosis and therapy. They thoroughly reviewed the outstanding properties of exosomes, especially those derived from prostate tumors, and mediating intercellular communications with stromal cells, as well as tumorigenesis, tumor progression, angiogenesis, metastasis, tumor immune escape and drug resistance. They also recapitulated the many exosomal components (proteins, RNAs and lipids) already demonstrated as putative candidates for improving the specificity of early PCa diagnosis and patients’ future prognosis.

After reviewing many methods for PCa diagnosis, Davey et al. [66] performed a pilot study, using Vn96 affinity captured EVs to provide mRNA and miRNA biomarkers for improved accuracy of prostate cancer detection. They enrolled 56 patients, which could not be discriminated by serum PSA testing, 28 negative and 28 positive for PCa in different clinical stages, based on tissue biopsy results. They isolated EVs from post-digital exam urine and identified a panel of seven mRNA biomarkers able to discriminate non-cancer from cancer with a sensitivity of 75% and specificity of 84%. They also identified two miRNAs, miR-375-3p and miR-574-3p, which, when paired with the seven mRNA panel, yielded a clinically applicable diagnostic test able to distinguish noncancerous conditions from prostate cancer with a sensitivity of 79% and a specificity of 89%. 

Although there has been extensive characterization of the protein and nucleic acid components of EVs, their lipidome has received little attention. Brzozowski et al. [67] performed thorough lipidomic profiling of EVs derived from non-tumorigenic (RWPE1), tumorigenic (NB26) and metastatic (PC-3) prostate cell lines. They identified differences in the molecular lipid species of prostate cell-derived EVs, increasing understanding of the changes that occur to the EV lipidome during PCa progression. This may lead to improved diagnostic and prognostic biomarkers for PCa. 

In 2017, Shi et al. [68] recapitulated the first trials using plasma (exosomal and protein-associated) RNAs to diagnose PCa, and the many challenges to solve before applying them in a non-invasive blood test. At the same time, Pucci et al. [69] suggested the use of exosomes in semen as a new tool in prostate cancer diagnosis. Seminal fluid contains a high concentration of EVs, discovered in 1982 and named “prostasomes” (with a diameter of 50 to 500 nm), released by prostate epithelial cells. The role of prostasomes in PCa is discussed, focusing on their possibility to offer a non-invasive test, complementing the poor specificity of the blood prostate antigen (PSA) biomarker for PCa diagnosis. Barceló et al. [70] extensively studied the expression level of miRNAs contained in semen exosomes from men with moderately increased PSA levels to assess their usefulness, either alone or in addition to PSA marker, as non-invasive biomarkers, for the early efficient diagnosis and prognosis of PCa. Fourteen miRNAs were selected for miRNA validation as PCa biomarkers. The authors described miRNA-based models which, together with PSA levels, improved the classification function of the PSA screening test with diagnostic and/or prognostic potential: [PSA + miR-142-3p + miR-142-5p + miR-223-3p] model (AUC: 0.821) to discriminate PCa frombenign prostatic hyperplasia (BPH); and [PSA + miR-342-3p + miR-374b-5p] model (AUC: 0.891) to discriminate between (Gleason Score) GS ≥ 7 tumors and men presenting PSA  ≥  4 ng/mL with no cancer or GS6 tumors.

Besides blood and semen, urine has also been used to isolate EVs for prostate cancer diagnosis. Shin et al. [71] performed a very interesting optimization of an aqueous two-phase system (ATPS) to isolate extracellular vesicles from urine for prostate cancer diagnosis. The ATPS isolation method optimized by adjusting (polyethylene glycol (PEG)/dextran (DEX)) polymer concentration recovered approximately 100% of EVs from the urine, whereas “U/C-once” (one 100.00× *g* 2 h) and “UC-twice” (two 100.000× *g* 2 h) ultracentrifugations, respectively recovered 21% and 6.85% EVs. Moreover, besides the high ATPS recovering efficiency, EVs are isolated within only ~30 min, and without the requirement for any special equipment, both of which are very clinically useful. Protein markers and RNAs were analyzed showing an easy detection of CD9, CD81 and CD63 EV surface markers in the EVs isolated by ATPS, whereas they were not detected with UC-twice due to their low isolation efficiency. The same was observed with RNAs. Afterward, the compatibility of these EVs with PCa diagnostic purpose was studied, using urine from 20 prostate cancer (PCa) patients and 10 (BPH) patients. PCa-derived urine exosomes being hypothesized to contain the two PCa biomarkers Prostate-specific membrane antigen (PSMA) and the PCA-specific gene marker (PCA3), these two EV components were chosen for checking the EV capacity for patients classification, with states otherwise identified by serum-PSA and tissue biopsy. The EV diagnostic ability based on ATPS isolation was better than other conventional methods and correctly identified all 10 PCa patients from the BPH patients, with improved sensitivity and specificity, when RNA and protein were combined.

For PCa, as for other cancers, the suggested EV-mediated liquid biopsies should be considered as interesting proofs of concepts, but there might be a long way before their efficient clinical translation, as using EVs as a potential source of cancer biomarkers in body fluids is still in its infancy. Many technological challenges remain to be solved such as promoting a clinical-valid method for isolating well-characterized EV subpopulations, and elaborating standardized protocols for appropriate large-scale multicenter studies of some most promising exosomal biomarkers. Some clinically relevant set-ups have already been described, but their review is out of the scope of this study. As a mere example of this current approach, the work of Cho, Yang and Rhee [72] might be mentioned. They developed in vitro for the first time a simultaneous multiplexed detection of exosomal microRNAs and surface proteins as a simple, cost-effective, noninvasive potential EV-mediated liquid biopsy for diagnosing PCa.

### 5.4. Colorectal Cancer EV-Mediated Diagnosis

Colorectal cancer (CRC) is the third most commonly diagnosed malignancy and the fourth most common cause of cancer-related death worldwide. The 5-year survival rate is highly stage-dependent and only 14% at stage IV, and even much less for the 40 to 50% of CRC patients developing metastatic disease. Approximately 20–25% had undergone liver metastasis at the time of CRC diagnosis. Therefore, methods for early diagnosis of CRC are urgently needed.

Recently, Vafaei et al. [73] reviewed liquid biopsy as an expanding non-invasive tool in the management of CRC patients in different stages, more specifically focusing on complementary circulating tumor cells (CTCs) and tumor-derived exosomes (TDEs). They compared the merits of CTCs and TDEs, but mentioned that further validations are required before addressing their putative applications in oncology. Herrera et al. [74] also concerned with liquid biopsy for CRC, focused on the components of the tumor microenvironment detected in blood samples of CRC patients. They performed an interesting extensive study about the crosstalk by exosome transfer between tumor and microenvironmental cells, such as cancer-associated fibroblasts (CAFs), Mesenchymal Stem Cells (MSCs), immune cells and vascular cells. They considered the respective exosomes, not only derived from tumor cells, but from all stromal cells involved in cancer progression. This review is a treasure chest for future potential biomarkers for CRC diagnosis.

So far, most studies have used 2D cell cultures to unravel the functions of EVs in CRC. The recently developed 3D technology represents a superior model that maintains the cellular heterogeneity of in vivo tumors without mesenchymal cells. Szvicsek et al. [75] studied the CRC organoid-derived EVs by using the SW1222 CRC cell line; then, they were the first to apply this technology for EV analysis by using direct CRC patient-derived samples. They found that EV release is induced from organoids after *Apc* mutation, a critical genetic event in CRC, and in the presence of collagen I, which often accumulates in CRC. Furthermore, fibroblast-derived EVs enhance the colony-forming ability of CRC organoid cells in hypoxia. These results highlight the power of the 3D technology and provide clues for using EVs as diagnostic markers in CRC.

Now, micro RNAs (miRNAs), a class of small non-coding RNAs of 19–22 nts, are widely considered for being potential new cancer biomarkers, especially for CRC. Other more recent studies also involved miRNAs [76,77,78]. In 2017, Zhu et al. [76] performed a four-phase study to screen miRNAs in CRC serum samples. Among 168 serum miRNAs, they identified a panel of three miRNA (miR-19a-3p, miR-21–5p and miR-425–5p) for the diagnosis of CRC. Their serum levels were significantly higher in patients with CRC than in NCs. Elevated expression of the three miRNAs was also observed in CRC tissues (*n* = 24). Furthermore, the expression levels of the three miRNAs were significantly elevated in exosomes from CRC serum samples (*n* = 10). Zhang et al. [77], also conducting a four-stage study, suggested a plasma seven-miRNA signature (miR-103a-3p, miR-127–3p, miR-151a-5p, miR-17–5p, miR-181a-5p, miR-18a-5p and miR-18b-5p) as potential CRC diagnosis. Additionally, miR-103a-3p, miR-127-3p, miR-17-5p and miR-18a-5p were significantly upregulated in CRC tissues, while miR-17-5p, miR-181a-5p, miR-18a-5p and miR-18b-5p were significantly elevated in CRC plasma exosomes. The two previous studies were mostly concerned with blood miRNAs as CRC biomarkers, although the corresponding miRNAs were also found, respectively in serum exosomes, and plasma exosomes. More directly related with EVs, Sun et al. [78] were the first to discover that elevated tumoral miR-122 released in serum was delivered by exosomes and to identify serum exosomal miR-122 as a potential diagnostic and prognostic biomarker of CRC with liver metastasis (LM). Serum exosomal mir-122 expressions could obviously discriminate CRC patients with LM from healthy individuals as well as CRC patients without LM. Moreover, CRC patients with higher circulating exosomal miR-122 expression suffered from unfavorable prognosis.

Recent studies mentioned long noncoding RNAs (about 200 nts) as potential novel diagnosis and prognosis biomarkers in CRC. In the last years, several studies searched for CRC diagnosis-efficient EVs-derived lncRNAs [79,80,81,82]. Hu et al. [79] isolated exosomes from the plasma of CRC patients (*n* = 50) and healthy individuals (*n* = 50) and performed exosomal lncRNA profiling in the two groups. The expression of six exosomal lncRNAs was significantly upregulated in the plasma of CRC patients and suggested as potential non-invasive biomarkers for early diagnosis of CRC. Wang et al. [80] started from the crucial role played by the lncRNA colon cancer-associated transcript 2 (CCAT2) in several cancers and questioned its clinical significance in CRC. They found that expression of CCAT2 was upregulated both in CRC tissues and in serum of CRC patients. Higher CCAT2 expression was associated with advanced CRC diseases and significantly decreased in post-operative samples. Then, they investigated the expression levels of CCAT2 levels in exosomes isolated from serum from CRC patients and healthy controls and observed that CCAT2 was contained in EVs with a diameter mostly within the range of 20–200 nm and showing the same properties as the whole serum. With the assumption that the circulating CCAT2 might be protected by exosomes, they suggested serum exosomal CCAT2 as a novel potential predictor in CRC. Oehme et al. [81] asserted that a low level of exosomal lncRNA HOTTIP is an independent valid prognostic biomarker in CRC to predict post-surgical survival time. Lastly, Yu et al. [82] focused on the lncRNA X inactive specific transcript (XIST) already shown to be overexpressed in CRC cell lines and tissues and with a high expression correlated with higher tumor size, stages, metastasis and adverse overall survival in patients. The authors identified small serum EVs and found for the first time that lncRNA XIST was highly expressed in serum EVs in CRC patients. Furthermore, lncRNA XIST expression was related to the malignant degree and a high lncRNA XIST expression predicted a worse prognosis. Moreover, they found a positive correlation between lncRNA expression and the serum CRC biomarkers CEA, CA242, CA199 and CA153 in the serum of the 94 patients under study. Thus, they convincingly demonstrated that serum-EV-transported lncRNA XIST has diagnostic and predictive effects on CRC.

Circular RNAs (circRNAs), single-stranded covalently closed RNAs, were recently reported as being closely associated with the initiation and development of cancers, including CRC. Pan et al. [83] identified the serum exosomal hsa-cir-0004771 as a potential novel CRC early diagnostic biomarker. The exosomal hsa-cir-0004771, originating from the tumor, was found significantly upregulated in the serum of CRC patients, compared to healthy controls and patients with benign intestinal diseases. Moreover, its expression was downregulated in the serum of post-operative CRC patients. 

### 5.5. Ovarian Cancer EV-Mediated Diagnosis 

Ovarian cancer is the leading cause of death among gynecologic malignancies and the fifth cause of cancer-related death among women. Since ovarian cancer develops asymptomatically, it is often diagnosed at an advanced and incurable stage. Until recently, the main diagnostic approaches for ovarian cancer included the blood biomarker CA125 and imaging. However, CA125 is not elevated in the early stage and not enough specific, stressing the urgent need for more efficient biomarkers. 

Nakamura et al. [84] reviewed the clinical relevance of circulating cell-free micro RNAs (cf-miRNAs) in ovarian cancer. Interestingly, they summarized the current knowledge about miRNAs in different body fluids for different human cancers (see Figure 1 [84]). Moreover for ovarian cancer, they took into account liquid biopsy studies in the blood (plasma/serum), ascites or urine, involving all the cf-miRNAs, not only those transported by microvesicles, exosomes, or apoptotic bodies, but also those protected by RNA-binding protein such as Argonaute 2, or lipoprotein complexes. This 2016 review, involving about 20 studies gives a nice preliminary overview of cf-miRNAs as potential diagnostic biomarkers of ovarian cancer. At the same time, Zhao et al. [85] developed a microfluidic ExoSearch chip for multiplexed exosome detection towards blood-based ovarian cancer diagnosis.

Hoping to provide new information on exosome implications in cancer diagnostic and treatment, Shen et al. [86] described the characteristics of exosomes in ovarian cancer, especially focusing on their role in immune modulation and drug resistance. Ovarian cancer-derived exosomes may be involved in tumor development, drug resistance and immune regulation (both immune suppression and stimulation) via transferring different bioactive proteins and miRNAs. The tumor cells–derived exosome cargoes are tumor-specific and correlate with tumor staging and prognosis. Compared with exosomes derived from benign ovarian lesions, exosomes from ovarian cancer contain significantly increased levels of TGFβ1 and melanoma-associated antigens MAGE3 and MAGE6, suggesting potential biomarkers for distinguishing malignancy from benignity. Previous studies showed also that the expression of miR21, miR-141, miR-200a, miR-200b, miR-200c, miR-203, miR205, and miR-214 was significantly elevated in ovarian cancer exosomes. As informative carriers between cells, exosomes have a promising value in early diagnosis and prognosis assessment.

Barnabas et al. [87] postulated that a liquid biopsy, such as utero-tubal lavage (UtL), might identify localized lesions better than systemic approaches of serum/plasma analysis for high-grade ovarian cancer (HGOC), which is diagnosed at a metastatic stage. They performed deep proteome profiling of the microvesicles of a total of 187 liquid biopsies and extracted a 9-protein classifier with high accuracy. The signature predicted all the early-stage lesions and outperformed the known markers CA125 and HE4 with 70% sensitivity and 76.2% specificity, revealing the potentiality of UtL-microvesicle proteomics for early diagnosis of HGOC. Chen et al. [88] investigated changes in CA125 and HE4 expression in serum-derived exosomes of 55 patients with OC (OC group), 33 patients with malignant tumors (non-OC group), and 55 normal controls (NC group). They also compared serum- and exosomal CA125 and HE4 levels. They found that CA125 can be detected at higher levels in exosomes but in all three groups, whereas HE4 was undetected in exosomes. Compared with CA125 or HEA alone, serum HE4+ exosomal CA125 combination improved OC diagnostic efficiency to 96.36% sensitivity and 92.7% specificity. 

Three recent reviews were devoted to liquid biopsies for ovarian cancer diagnosis [49,89,90]. Chang et al. [89] discussed the growing popularity of liquid biopsy versus tissue biopsy and the advantages of EVs for liquid biopsy, when compared to CTCs and ctDNA. They summarized the potential EV protein biomarkers in ovarian cancer diagnosis, prognosis and therapy (see Table I [89]). They also highlighted the utility of new technologies recently developed for EV detection with an emphasis on their use for diagnosing ovarian cancer, monitoring cancer progression, and developing personalized medicine. The clinical significance of CTCs and ctDNA in ovarian cancer has been intensively investigated in a large number of research studies over the last two decades. Giannopoulou et al. [90] provided a brief description of the most recent studies on CTCs and ctDNA and mainly focused on the clinical potential of circulating cell-free and exosomal miRNAs in ovarian cancer. They presented in detail many studies involving more than 25 ovarian cancer patients (see Tables I–III [90]) and reporting many over-expressed or under-expressed miRNAs in ovarian cancer patients when compared to healthy controls. They checked their diagnostic value, but the big amount of results was sometimes controversial and not conclusive. The first study on circulating exosomal miRNAs in ovarian cancer was performed in 2008. A large number of exosomal miRNAs were examined, but only eight (miR-21, miR-141, miR-200a, miR-200c, miR-200b, miR-203, miR-205, and miR-214) were found elevated both in serum samples and paired primary tumors. These miRNAs were not detected in healthy controls and patients with benign disease, indicating a possible clinical value of these circulating exosomal miRNAs in the early diagnosis of ovarian cancer. A more recent study mentioned four miRNAs (miR-373, and also miR-200a, miR-200b, and miR-200c), as significantly overexpressed in ovarian cancer patients compared to healthy controls. Interestingly, the increased levels of miR-200b and miR-200c were also significantly correlated with the CA125 biomarker levels. Lastly, Norouzi-Barough et al. [49] questioned the potential of circulating tumor-derived exosomes (TDEs) in the diagnosis and prognosis of ovarian and breast cancers. Concerning ovarian cancer, they performed a very interesting analysis of six studies involved in exosomal protein profiling (from 2009 to 2016) and of six other studies concerned with the identification of exosomal micro RNAs (from 2008 to 2016). Except for one in vitro study concerned with two low or high OV cell lines, all the studies dealt with different OC patient’s biofluids (mostly serum or plasma, but also effusion supernatants, urine and ascites). They managed to establish a classification of the clinical value of the different discovered exosomal biomarkers, convincingly discriminating different stages of OC patients from benign OC patients and healthy controls, more specifically with a few exosomal cf-miRNAs (see Table 1 [49]). This last review strongly emphasizes the promising assets of TEDs for early diagnosis of ovarian cancer, as well as after-treatment monitoring and even therapy. However, many technological issues remain to be solved, and it might still be a long way ahead before an efficient clinical translation is achieved. 

### 5.6. Pancreatic Cancer EV-Mediated Diagnosis

Pancreatic adenocarcinoma (PaC) ranks fourth in mortality among cancer-related deaths. With an overall 5-year survival rate of below 1% and a mean survival time of 4–6 months, it is the deadliest cancer [91]. Traditional biomarkers such as carcinoembryonic antigen (CEA) and cancer antigen 19–9 (CA19–9) have improved the diagnostic accuracy of pancreatic cancer, but with a low specificity because of high CA19–9 expression in benign pancreatic diseases and increased CEA expression in colorectal cancer [92].

Recently, Mountinho-Ribeiro et al. [93] presented an interesting state-of-the-art about PaC diagnosis. Following their suggestion that the initial mutation in PaC might appear nearly 20 years before any symptom occurs, it is worth seeking biomarkers for early PaC diagnosis. However, conventional diagnostic tools are insufficient for early detection. Liquid biopsy offers a new horizon for PaC early detection and survival improvement. Besides other tumor markers already much involved in liquid biopsy, such as circulating tumor cells (CTCs) and cell-free DNAs (cfDNAs), exosomes are now coming onto the stage as interesting candidates for early PaC diagnosis. Zhu et al. [94] performed a systematic comparative review of various liquid biopsy methods for pancreatic cancer. They found that liquid biopsy was a powerful test for PaC detection and that exosomes had the highest overall diagnostic value, with high sensitivity and specificity. Among the three subtypes of liquid biopsy, CTCs were the less valuable, ctDNAs were suitable for diagnosis only, and exosomes were the best ones for both PaC diagnosis and screening.

Earlier, Lorenzon and Blandino wrote a commentary with preliminary reports about circulating exosomes in body fluids of patients with different cancers [95]; they principally focused on Glypican-1 exosomes mainly associated with PaC and wondered whether this might initiate a new era for early pancreatic cancer diagnosis.

In 2019, Zou et al. [96] identified by qRT-PCR a promising six-miRNA panel (let-7b-5p, miR-192–5p, miR-19a-3p, miR-19b-3p, miR-223–3p, and miR-25–3p) in serum for improving PaC early and noninvasive diagnosis. In addition, significant upregulation of miR-192-5p, miR-19a-3p, and miR-19b-3p was observed in both PaC tissue and serum-derived exosomes samples, whereas increased serum miR-19a-3p was closely related to worse overall survival (OS). At the same time, Pang et al. [92] developed a dual-SERS biosensor for one-step detection of microRNAs in exosomes and residual plasma of blood samples for diagnosing PaC. The intercellular communication function of exosomes is mediated by their complex cargo composition of a variety of bioactive molecules (such as proteins, lipids and miRNAs), which can be transferred from donor cells to recipient cells for orchestrating the physiological processes. When released from tumor cells, both the concentration of tumor exosomes in body fluids is notably increased and their cargo composition is modified, when compared to normal cell-derived exosomes. Therefore, the choice of the best exosomal biomarkers for cancer diagnosis is rather challenging and still under worldwide study.

For PaC, the search for exosomal biomarkers is still in its infancy. Zöller compared PaC diagnosis by free and exosomal miRNA and nicely advocated for the use of exosomal miRNAs [91]. One argument was that exosomal tumoral miRNAs were derived from live tumor cells contrary to free miRNAs, associated with Argonaute2 protein and derived from dead cells; moreover, exosomes offer the possibility to improve the miRNA diagnosis by joining specific membrane protein biomarkers for diagnosis. This was confirmed by Madhavan et al. [97] who suggested an association of a panel of proteins from a small population of PaC initiating cells (PaCIC) and miRNA serum exosome biomarkers for increasing sensitivity and specificity for PaC diagnosis. Protein markers were selected according to expression in exosomes of PaC cell line culture supernatants, but not in healthy donor’s serum-exosomes; miRNA were selected according to abundant recovery in microarrays of patients with PaC, but not in healthy donors’ serum-exosomes and exosome-depleted serum. Serum-exosomes were tested by flow cytometry for the PaCIC markers CD44v6, Tspan8, EpCAM, MET and CD104. Both serum-exosomes and exosomes-depleted serum were tested for miR-1246, miR-4644, miR-3976 and miR-4306 recovery by qRT-PCR. The majority (95%) of (131) patients with PaC and patients with nonPa-malignancies reacted with a panel of anti-CD44v6, -Tspan8, -EpCAM and -CD104. Serum-exosomes of healthy donors and patients with nonmalignant diseases were not reactive. Recovery was tumor-grade and staging-independent including early stages. The selected miR-1246, miR-4644, miR-3976 and miR-4306 were significantly upregulated in 83% of PaC serum-exosomes, but rarely in control groups. These miRNA were also elevated in the exosomes-depleted serum of patients with PaC, but at a low level. Concomitant evaluation of PaCIC and miRNA serum-exosome marker panels significantly improved sensitivity (1.00, confidence interval CI: 0.95–1) with a specificity of 0.80 (CI: 0.67–0.90) for PaC versus all others groups and of 0.93 (CI: 0.81–0.98) excluding nonPa-malignancies.

## 6. Challenges for Clinical Translation of EV-Mediated Cancer Diagnosis 

The huge amount of scientific publications concerned with EVs as potential appealing biomarkers in liquid biopsy for cancer diagnosis has to be moderated by the many challenges to solve for their efficient clinical translation. Inasmuch as each cancer has its own specificity, with regard to the initiation, genetic and epigenetic dysregulations and time course of progression, it might be more appropriate to consider each of the 36 human cancers taken into account by the GLOBOCAN cancer statistics [1] as separated diseases for diagnosis. All the rather recent publications reported in this review have to be considered as preliminary studies aimed to discover the most interesting EV biomarker or panel of EV-transported biomarkers for a specific human cancer. This search is only in one’s infancy and should keep on ongoing, inasmuch as the golden tumor biomarkers might still be hidden in the treasure chest of the EV cargoes. Each of these preliminary approaches is now most often followed by searching a kind of pre-clinical validation among a small number of patients suffering from this specific human cancer at various stages of the disease compared with healthy controls, in order to define the specificity and sensitivity of the EV biomarkers in consideration. Besides that, it is strongly required to define a robust clinical protocol aimed at standardizing isolation and characterization of EVs, as well as the clinical tests for screening, testing and validating the most promising EV biomarkers, among many more patients with well defined clinical characteristics and suffering from one of the main specific human cancers. This essential step is a prerequisite remaining to fulfill further strong multicenter investigations, aimed to classify the interest of the many potential EV candidates for achieving efficient early detection of any specific human cancer. Indeed, this might still be a long-term work, as detailed by Zhao et al. [98], discussing approaches for EV biomarker discovery and verification, EV clinical assay development, analytical and clinical validations, clinical trials, regulatory submission, and end-user utilization for the intended clinical application.

## 7. Conclusions

Liquid biopsy has been a main technological advance in cancer diagnosis when compared with the much more limited and invasive tissue biopsies. Whereas liquid biopsy first relied during many years on blood circulating cancer cells (CTCs), then on circulating cell-free DNAs (cfDNAs), circulating exosomes recently appeared as a third important player in liquid biopsy. This review shows the different steps of the EV/Exosomes-mediated early cancer diagnosis successively relying on various components of the rich exosomal cargoes. By comparison with the long-lasting research about the two previous liquid biopsies, EV-mediated liquid biopsy is still in its infancy and cannot yet be efficiently compared with CTCs and cfDNA-mediated liquid biopsies.

The present original literature search about EV-mediated cancer diagnosis by using the Expernova Questel platform brought many interesting reviews and articles. Among the easily accessible 264 scientific papers, a two-part selection was performed. Some of the most pertinent recent scientific papers (2019–2020) were first chosen in order to give a general insight into the current trends of EV-mediated cancer diagnosis in liquid biopsy. In the second part, pilot studies about EV-mediated cancer diagnosis for six specific human cancers (lung, breast, prostate, colorectal, ovarian and pancreatic) were chosen to illustrate the first preclinical trials towards clinical applications. The main goal of this review was to advocate the huge complementary interest of EVs for future non-invasive early diagnosis of cancer among medical readers not yet familiar with this new topic. However, many technical challenges remain yet to be solved before the necessary multicenter validation of some of the already promising candidates as specific cancer biomarkers, and this might still be a long-term work [98]. However, the assets of EVs with regard to both the already known EV composition and their important epigenetic biological functions are worth further worldwide investigations. This might allow reaching a positive demonstration of the EV potential for becoming outstanding biomarkers for early cancer diagnosis in the future. Another important challenge might be to understand the huge heterogeneity of the EV continuum with their respective regulated macromolecular cargoes equipped for intercellular communication. This would allow classifying the different EV subpopulations as a function of their respective biological functions. Then, one might further imagine that a few cancer-specific EVs subpopulations, concentrating many of the presently discovered exosomal cancer biomarkers, might be specifically discriminated from the whole EV subpopulations orchestrating all the biological functions needed for health. 

## Data Availability

Data available through each URL obtained by using the Expernova Questel platform.

## Supplementary Materials

The following are available online at https://www.mdpi.com/article/10.3390/ijms22115674/s1. Some details about the literature search by using the Expernova Questel platform will be given in the supplementary files.
