# Supplementary material for: Current Search through Liquid Biopsy of Effective Biomarkers for Early Cancer Diagnosis into the Rich Cargoes of Extracellular Vesicles
Source: Int J Mol Sci. 2021 May 26;22(11):5674. doi: 10.3390/ijms22115674 (PMC8199101; doi:10.3390/ijms22115674)
Supplement: Supplementary file 1 [file ijms-22-05674-s001.zip › ijms-1188272-supplementary.pdf]

International Journal of *Molecular Sciences*

MDPI

Supplementary Materials

Literature search through the Expernova-Questel *big data* platform

The *big data* platform was elaborated by-the french start-upExpernova, founded in 2008 and based in Montpellier. The aim was to optimize the choice of participants in any projects covered by the 400 domains of the platform. After watching two webinars given by E. Naudi, it became clear that it would be a wonderful tool for searching worldwide EV-dedicated literature.

A first quick comparative search in Advanced PubMed and Web of Science was performed with the search terms "Cancer Diagnosis" AND "Extracellular Vesicles" with a nine year (2012–2020) or a five year (2016–2020) filter. The search brought respectively 141 and 134 scientific papers (SPs) with PubMed and 238 and 224 with Web of Science, showing that the topic became rather hot in the last five years. However, the results of such bibliographic search are very dependent on the search terms. Performing the same five years search, but taking into account the known whole diversity of extracellular EVs (i.e. "Sum EVs" defined below) gave as much as 331 references with PubMed and 384 with Web of Science. These two databases are indeed very convenient for a well-defined research with a rather small number of results, but their use becomes really cumbersome when dealing with a few hundred SPs.

For the current literature search, the Expernova Questel platform was used with the first search term, "Cancer Diagnosis" AND the second term (Microvesicles OR Exosomes, OR Apoptotic Bodies OR Oncosomes, OR "Extracellular Vesicles"), corresponding to "Sum EVs" in order to better cover the whole EV continuum, and without any year range filter. Changing the respective orders of the two terms or of each component inside the bracket gives the same results. Figure S1 shows the typed search and the six overall results obtained "in one click", pointing out 374 scientific papers (SPs). Figure S2 gives a detailed overview of the obtained results. This can be exported as a pdf (9p), or as a 01--image.png, corresponding to the figure. It may also be exported as a (package).zip (3.4 Mo), expanding to all the different parts of the overview (41.7 Mo), as described beside Figure S2.

Among the obtained 374 SPs, 264 were found to be either open or easily accessible through the UPMC online library. All SPs from the ongoing search were easily exported as Excel tables, with thirteen specific data per SP (i.e. Number, Title, Abstract, Keywords, Domains, Language, Publication type, Reviewed, Source Name, SourceURL, Authors, Authors Affiliations, Year). From the four exported Excel tables, corresponding Word tables were prepared by keeping only the Number, the Title, the Abstract, the Source Name (PubMed Central, PubMed from Medline, Medline, CrossRef or Doaj), the two first mentioned Authors, the Source URL, and the Year of publication. Live on the platform everything was interactive and when clicking on a given SP, a lot of complementary informations could be accessed. The different SPs could also be exported as pdf files as shown on Figure 3 for the first five references, but with loss of complementary informations.

For the present review, the whole references of the literature search were obtained from the Expernova Questel platform within one day. The criteria for further SP selection relied mainly on the title, abstract and year of publication, followed by complete reading of each of the corresponding telecharged pdf.

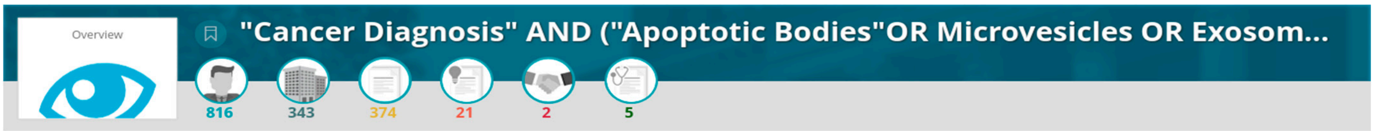

Figure S1. Search through the Expernova Questel platform for the present review. From left to right the numbers correspond to the 816 people, the 343 private companies and academic institutions, the 374 scientific papers, the 21 patents, the 2 projects and the 5 clinical trials, which are involved worldwide by this search.

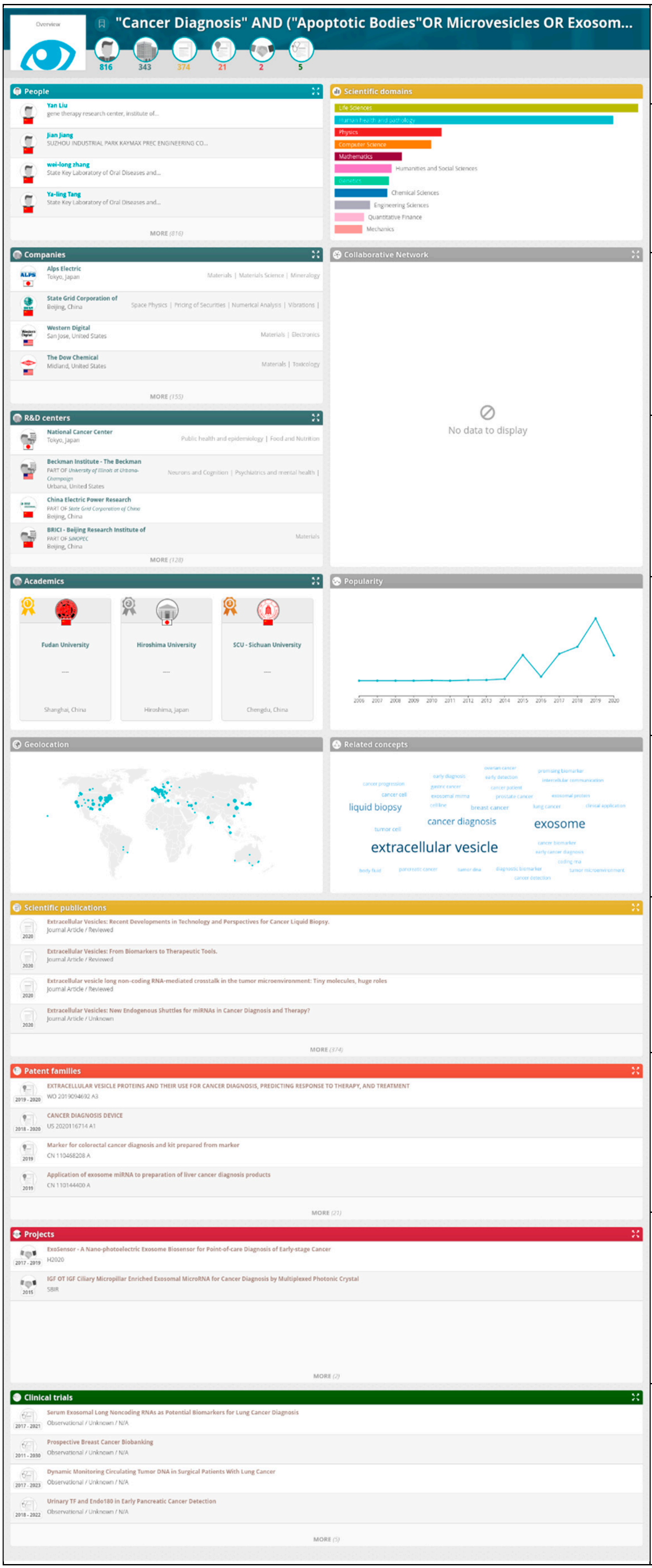

Content of the Overviewpackage	
02-People.png	001-scientific domains.png
03-Companies.png	Collaborative Network
04-R & DCenters.png	
05-Academics.png	002-image-export.png *(Popularity)*
003-image-export.png *(Geolocation)*	004-image-export.png *(Related concepts)*
**06-Scientific-Publications.png**	
07-Patent-Families.png	
08-Projects.png	
09-Clinical-Trials.png	

Figure S2. Overview of the results obtained "in one click" on 16/12/2020 at 10:41 with the corresponding Content of the Overview package.

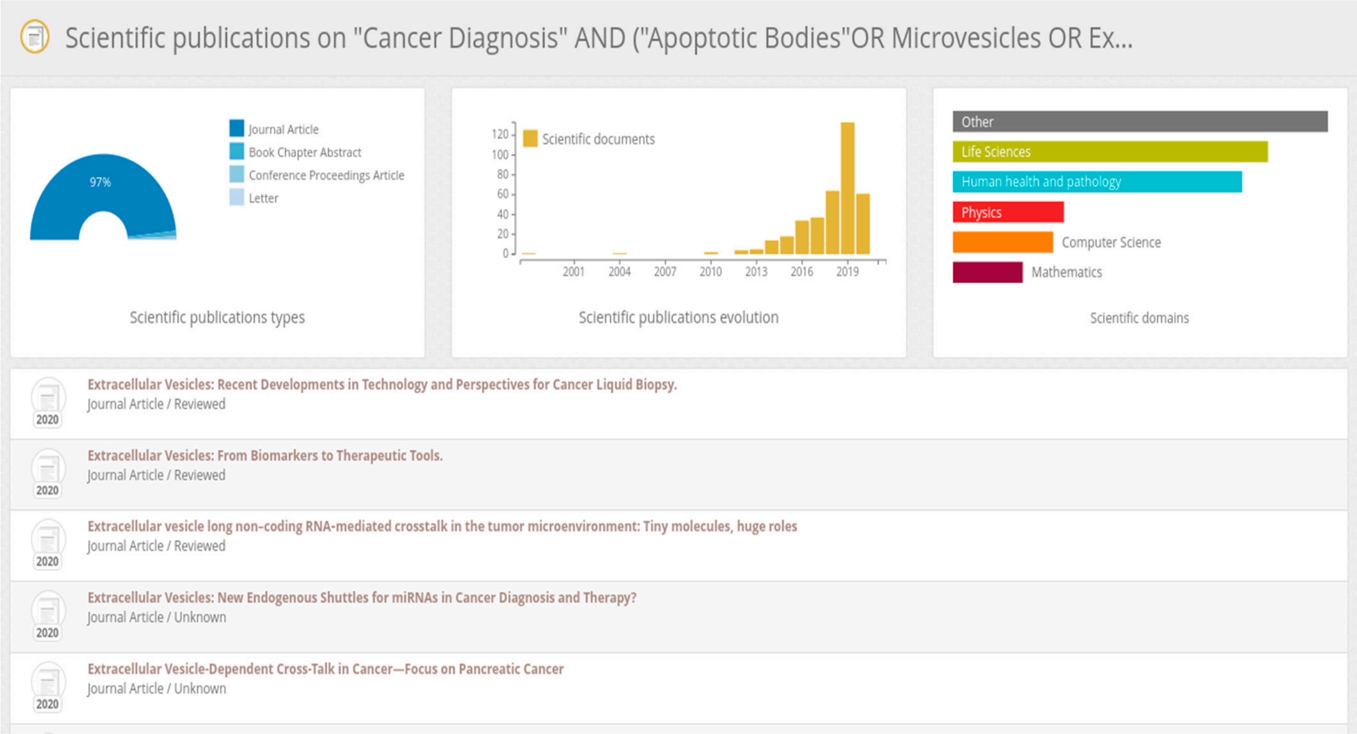

Figure S3. First five Scientific Publications exported in pdf.
